# Gut Microecology of Four Sympatric Desert Rodents Varies by Diet

**DOI:** 10.1002/ece3.70992

**Published:** 2025-02-27

**Authors:** Dongyang Chu, Haoting Zhang, Zhenghaoni Shang, Nan Liu, Heping Fu, Shuai Yuan

**Affiliations:** ^1^ College of Grassland, Resources and Environment Inner Mongolia Agricultural University Hohhot China; ^2^ Key Laboratory of Grassland Rodent Ecology and Rodent Pest Control at Universities of Inner Mongolia Autonomous Hohhot China; ^3^ Key Laboratory of Grassland Resources of the Ministry of Education Hohhot China

**Keywords:** dietary strategy, gut microbiome, rodents, sympatric coexistence

## Abstract

The gut microbiome can be one pathway by which a host rapidly acclimates and adapts to its ecological environment. Exploring how the microbiome has evolved to differ between hosts with different diets provides insights into the profound interactions between hosts and microbes within these systems. In this study, we used DNA metabarcoding techniques and macrogenomic prediction techniques to study the gut microbes of four desert rodent species with different feeding strategies in the same habitat. One species is herbivorous (*Spermophilus alashanicu*)*s*, one is granivorous ( *Phodopus roborovskii* ), another is omnivorous ( *Dipus sagitta* ), and the last (*Orientallactaga sibirica*) has a diet with a relatively high proportion of insects. Diets rich in plants and insects can be challenging to digest due to the abundance of indigestible fiber and stable chitin, respectively. Out of the species studied, the herbivorous *Spermophilus alashanicus* has the highest density of *UCG‐005* genes and the highest predicted abundance of genes related to digestive complexity. The composition of *Phodopus roborovskii* 's microbiome has the highest variation between individuals, yet *Phodopus roborovskii* has the highest predicted abundance of genes associated with simple sugars—reflecting this species' potential adaptability to the starch within plant seeds and its constraints brought about by its smaller body size. The most insectivorous species, *Orientallactaga sibirica*, exhibits the highest predicted abundance of genes related to chitin degradation. This study has enhanced our understanding of the gut microbiota in the intestines of rodents as they adapt to various dietary strategies.

## Graphical abstract

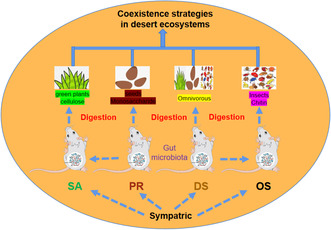

## 1 Introduction

The animal gut is one of the major sources of microbial biodiversity on Earth (Thompson et al. 2017), and the animal gut microbiome is a particularly dense and diverse community that co‐encodes 150 times more genes than its host (Qin et al. 2010). The composition of gut microbial communities is shaped by a complex set of host and environmental factors, including host genotype, species and individual development, diet, habitat, geographic location, and anthropogenic disturbance (Amato et al. 2013). While geography, sex, reproductive status, and social structure have all been associated with animal gut microbiome diversity, the main factors that consistently drive it appear to be host evolutionary history and diet (Maurice et al. 2015; Groussin et al. 2017; Pascoe et al. 2017).

The gut microbiome, heralded as “the second genome” for animals, affects the host in a wide variety of ways. Gut microbes carry out various physiological functions for the host, including the digestion and absorption of nutrients, energy metabolism, immune responses, signal conveyance, and the breakdown of toxins (Ursell et al. 2014; Canfora et al. 2015; Xu et al. 2019). Therefore, the gut microbiome has been proposed as one pathway through which the host organism can rapidly adapt to its ecological environment (Henry et al. 2021). These microbial communities are vital for degrading complex biological polymers or toxins that are indigestible by animals, such as the lignocellulose found within plant cells walls and substances like cellulose, lignin, and guaiac acid (Moura et al. 2010). Therefore, many species of herbivorous mammals depend on their gut microbiota for their metabolic processes, subsequently absorbing the meta‐products of this metabolic activity. Most studies indicate that herbivorous mammals possess a higher abundance of genes related to cellulose degradation compared to species with other diets (Zhu et al. 2011). These studies are conducted across various taxonomic groups by comparing related species that live in similar ecological habitats but which have different diets. The analysis of the differences between their microbiomes provides insights into the symbiotic relationships between hosts and their microorganisms.

To better understand the unique roles of gut microbiota in various feeding behaviors of animals, it is essential to further explore their adaptive contributions to the breakdown of complex biopolymers. Gut microbiota not only play a critical role in the decomposition of cellulose in herbivorous animals, but they also exhibit significant physiological functions in other specialized feeding strategies. Chitin is a polysaccharide compound primarily found within the exoskeletons of arthropods. The polymer is composed of N‐acetylglucosamine and D‐glucose. Chitin is a linear chain of polymers with resilience and biodegradability, and it is so common in nature that it is second only to cellulose globally in material abundance, so it is common in the diet of numerous vertebrates (Tang et al. 2015; Khattak et al. 2019). Some studies suggest that animals consuming chitin‐rich foods may form a symbiotic relationship with chitin‐decomposing bacteria, facilitating the digestion of their prey. For example, the convergently evolved chitinases found in various species, such as *Pipistrellus nathusii* , *Callithrix pygmaea* , and *Orycteropus afer* , indicate a convergence in their gut microbiomes (Teullet et al. 2023). However, the functions of these gut microbial communities and their potential role in digesting chitinous exoskeletons remain largely unexplored (Delsuc et al. 2014). Rodents are particularly suited to addressing these pivotal questions about host–microbe interactions. They are a particularly diverse order of mammals, with a rich array of ecological strategies. These animals have adapted to a multitude of different habitat types and exhibit a wide range of dietary strategies, including herbivore, granivore, omnivory, and insectivore (Kohl et al. 2014; Popov et al. 2023). Therefore, rodents have emerged as a ubiquitous model in evolutionary ecology, particularly in the investigation of the host‐gut microbiome interactions and how these relate to environmental factors (Weinstein et al. 2021). Although previous studies have investigated the microbiological adaptations of herbivorous rodents, there has been no investigation into how the gut microbiome varies between rodent species that live in the same region but have distinct diets (Moeller and Sanders 2020; Kohl et al. 2022). In this study, we selected four sympatric desert rodent species: the herbivorous *Spermophilus alashanicus* , which belongs to the Rodentia, Scuiromorpha, Sciuridae; the granivorous *Phodopus roborovskii* , which belongs to the Rodentia, Myomorpha, Cricetidae; the omnivorous *Dipus sagitta* , which belongs to the Rodentia, Myomorpha, Dipodidae; and the insectivorous *Orientallactaga sibirica*, which also belongs to the Rodentia, Myomorpha, Dipodidae. Notably, the *Spermophilus alashanicus* is the only species among these desert rodents that belongs to the Scuiromorpha and is also the largest in body size among the four desert rodent species.

Of the numerous techniques available for studying microbial communities, the most common approach involves sequencing the genomes of microbial DNA, namely by amplifying and decoding the 16S rRNA genes of bacteria and then generating a catalog of microbes present (D'Amore et al. 2016; Smets et al. 2016). This method is known as “metagenomic sequencing,” and it is a technique dedicated to the investigation of the microbial DNA sequences within an entire ecological community. The sequencing of genomic DNA, specifically that focusing on the 16S rRNA gene, is limited to specific genetic regions. In contrast, metagenomic sequencing assesses the entire genome, encompassing genes and their functions from all microbial species; however, such sequencing is exorbitantly costly, leaving researchers often constrained by financial limitations (Fadiji and Babalola 2020). Therefore, researchers have developed tools like Tax4FUN, Piphillin, PICRUSt, and PICRUSt2 (an upgraded version of PICRUSt that augments the precision and scope of function predictions) to generate a predicted functional database (the predicted metagenome) based on 16S rRNA databases. These technologies are suitable for the preliminary analysis of the predicted abundance of functional categories across various samples, yet they bear limitations due to the potential for uncharacterized microbial taxa within sequencing results derived from 16S rRNA (Douglas et al. 2020).

In this experiment, we employed DNA metabarcoding techniques and predictive metagenomic techniques to investigate the gut microbiota of four rodent species with distinct diets. These four murine species coexist in the same bioregion but have distinct feeding ecologies (Li 2023) (Table 1 and Table S1). We hypothesized that due to their different diets, these four species of rodents would possess unique microbial communities (Anderson et al. 2012). Specifically, we predicted that herbivorous rodents would possess the highest microbial diversity, akin to what is observed in other herbivorous mammals (Zoelzer et al. 2021). Finally, we hypothesized that the microbial taxa or functions present in a particular species'gut would correspond to the nutritional components of that host's diet. For instance, herbivores would have microbes that break down cellulose, and insectivores would have microbes that degrade chitin. We anticipated that the predicted abundance of genes associated with cellulose degradation would be highest within the herbivores *Spermophilus alashanicus* , and that the genes related to chitin degradation would be most plentiful among the most insectivorous host, *Orientallactaga sibirica* (predicted based on the PICRUSt2).

**TABLE 1:** Characteristics of study species.

Species	Order	Taxonomic family	Group	Sample size (*n*)♂:♀	Average weight(g)	Diet
*Spermophilus alaschanicus*	Scuiromorpha	Sciuridae	SA	3:4	165.89	Plant foliage, 85%–92%, Seeds, 3%–9%, insects,0%–7%
*Phodopus roborovskii*	Myomorpha	Cricetinae	PR	5:5	14.99	Plant foliage, 1%–8%, Seeds, 88%–95%, insects,1%–9%
*Dipus sagitta*	Myomorpha	Dipodidae	DS	5:5	67.89	Plant foliage, 57%–81%, Seeds, 11%–34%, insects,3%–13%
*Orientallactaga sibirica*	Myomorpha	Dipodidae	OS	5:5	95.73	Plant foliage, 21%–51%, Seeds, 18%–42%, insects,27%–51%

*Note:* Based on the dietary composition of the four rodent species, we categorized them as herbivorous (SA), granivorous (PR), omnivorous (DS), and insect‐dominant (OS), the same below.

## 2 Methods

### 2.1 Rodent Collection

In August 2021, four rodent species were collected from the desert area located in the southern part of Alashan Left Banner, Alashan League, Inner Mongolia Autonomous Region, China (37.887497° N, 105.381715° E). Among them were 7 SA, 10 PR, 10 DS, and 10 OS (Table 1). In the sampling area, we used live‐trap cages to capture rodents(we only selected adult individuals with normal reproductive status for sampling), using fresh peanut rice as bait. Traps were deployed early in the morning and checked in the evening and then again early the subsequent morning. It has been previously shown that the consumption of bait in live‐trap cages does not significantly affect the gut microbiome of rodents over a short period of time (McCleery et al. 2022).

Rodents captured in live‐trap cages were humanely euthanized, and rodent tissue samples were collected immediately afterwards. Cecal contents were stored in 2 mL sterile cryostat tubes, and samples were snap‐frozen in liquid nitrogen immediately after sampling and then stored at −80°C.

We analyzed the diet composition of four desert rodent species using the method of stomach dissection and visual observation (Yong et al. 2011). The components of stomach content samples were classified into three categories: (1) Plant stems and leaves: The chyme appears green and contains visible chunks, fibrous pieces, or granular residues of plant stems and leaves; (2) Plant seeds: The chyme appears pasty, usually white, milky white, or pale yellow, with identifiable fragments of seed coats; (3) Insects: The chyme is multicolored, with visible fragments of insect wings, legs, and larval skins. The stomach samples collected from the rodents were dissected, and bait components were removed from the stomach contents. (Peanuts were used as bait, which are not naturally available in the rodents' habitats and were easily identifiable, as the bait was typically located in the anterior part of the stomach. Additionally, in some stomach content samples, no bait was found because some bait had fallen near the live traps without being consumed, and in certain cases, the bait was stored in the cheek pouches of *Phodopus roborovskii* ). After removing the bait, the remaining stomach content samples were spread on graph paper, and the proportion of each component in the chyme was estimated based on the number of 1 cm^2^ grids occupied by each component.

Although several rodents were collected from slightly different locations in the desert zone, differences in gut microbial communities between rodent species are much greater than geographic differences (Ge et al. 2021; Smith et al. 2015), and our sampling areas were not far from each other. Furthermore, changes in microbial composition caused by differences in collection or storage procedures are even smaller than individual differences (Franzosa et al. 2014; Hang et al. 2014), so these factors do not strongly influence our results.

### 2.2 DNA Extraction

DNA was extracted from the cecum contents using the FastDNA Spin Kit for Soil DNA extraction kit following the manufacturer's instructions. We chose the cecum contents because the cecum has the highest microbial density and activity (Kohl et al. 2014; Yang et al. 2018). The extracted DNA was stored on dry ice and transported to Shanghai Meiji Biomedical Technology Co. The V3–V4 region of the bacterial 16SrRNA gene was amplified by PCR using the upstream primer 338F (ACTCCTACGGGGAGGCAGCAG) and the downstream primer 806R (GGACTACHVGGGGTWTCTAAT) (Li, Dai, and Mu, 2022; Nguyen 2017), and the resulting amplicon products were combined and purified. Libraries were constructed using NEXTFLEX Rapid DNA‐Seq Kit and sequenced to generate 2 × 300 base‐paired end reads. Detailed methods for amplification, library preparation, and sequencing are described in the Supporting Information S1. Sequence reads have been deposited in the NCBI database at (PRJNA1083208).

Quality control of the raw sequences was performed using fastp (https://github.com/OpenGene/fastp, version0.20.0) software (Chen et al. 2018). Splicing was performed using FALSH (http://www.cbcb.umd.edu/software/flash, version1.2.7) software (Magoč and Salzberg 2011). We filtered the bases with a quality value below 20 at the end of the reads, set a window of 50 bp, and truncated the back‐end bases from the beginning of the window if the average quality value within the window was below 20. We filtered the reads below 50 bp after quality control and removed the reads containing N bases. According to the overlap relationship between PE reads, we spliced pairs of reads into one sequence. The minimum overlap length was 10 bp, and the maximum mismatch ratio allowed in the overlap region of the spliced sequences was 0.2. We screened the non‐compliant sequences based on the barcodes at the beginning and end of the sequence, with a mismatch ratio of 0.2. The barcode and primers at the beginning and end of the sequence distinguished the samples and adjusted the sequence orientation. The allowed mismatch number of the barcode was 0, and the maximum primer mismatch number was 2. Using UPARSE software (http://drive5.com/uparse/, version 7.1), sequences were OTU‐clustered based on 97% similarity, and any reads identified as archaea, chloroplasts, or mitochondria were removed from further analyses (Edgar 2013; Stackebrandt and Goebel 1994). Species classification was annotated for each sequence using the RDP classifier (http://rdp.cme.msu.edu/, version2.2), comparing it to the Silva16SrRNA database (v138) with a comparison threshold set at 70% (Wang et al. 2007). In this experiment, we also followed the recommended protocol of extracting samples in mixed batches, so that any contaminants should be evenly distributed in the samples and therefore not lead to contamination being the cause of any observed species differences (Eisenhofer et al. 2019).

### 2.3 Ethics Statement

This study was conducted in accordance with the guidelines issued by the Ethics Committee for Animal Care and Treatment of Inner Mongolia Agricultural University. The committee requires all researchers and students associated with wildlife and laboratory animals to be certified in accordance with the requirements of the Ethics Committee of Inner Mongolia Agricultural University (NND2017012 and NND2022093).

### 2.4 Statistical Analysis

We utilized QIIME2 software to generate abundance tables for different taxonomic levels. We employed the Uparse software for OTU clustering and used it to conduct statistical analysis on OTUs to discern both shared and unique OTUs among the different rodent species. To compare the diversity of gut microbial communities across various species, we used the Mothur software to compute several metrics of alpha diversity for the gut microbiomes. Subsequently, we applied the Kruskal‐Wallis test and post hoc inquiries to test for differences in these indices among the different rodent species. Subsequently, we employed Bray‐Curtis (Bray and Curtis 1957) and the UniFrac metrics (Lozupone and Knight 2005) (both with and without weighting) to compute the difference values between all sample pairs, thereby comparing the community composition of the gut microbiomes across these rodent species. The Non‐metric Multidimensional Scaling (NMDS) diagrammatically represents the principal component analysis, demonstrating the interconnectedness of the gut microbiota. We test for microbiome differences using two methods: the Permutational Analysis of Multivariate Dispersions (PERMDISP) test within QIIME2 (999 arrangements) and the pairwise Bray‐Curtis distances calculated for inbreeding samples, with the Kruskal‐Wallis test and post hoc analyses used for conducting more detailed comparisons. Ultimately, we employed PERMANOVA within QIIME2 to conduct a statistical comparison of the microbial community composition between the rodent species.

To discern the ways in which the bacterial communities of rodent species differ based on their diets, we employed the Analysis of Composition of Microbiomes (ANCOM) difference test within QIIME2, comparing the abundances of specific phyla and genera. Before the detection of diversity abundance, we filtered out all bacterial taxonomic groups with read counts of less than 20, thereby eliminating the rare categories (Weinstein et al. 2021).

We used the QIIME2 plugin PICRUSt2 (Douglas et al. 2020) to generate a predicted functional list from our 16S rRNA database (predicted metagenome). We excluded any OTUs whose Nearest Sequenced Taxon Index (NSTI) values exceeded 2.0 during this process. Considerations regarding the predictive power of metagenomic modeling require a focus on prior knowledge, particularly with regard to the comparison of functional categories associated with biopolymer degradation.

By pulling the glycoside hydrolase subset from the carbohydrate activity enzyme database (Lombard et al. 2014) with the Kyoto Encyclopedia of Genes and Genomes (KEGG) entity database, we searched for pathways that are associated with our predicted results. We selected and analyzed the genes associated with simple sugars: α‐glucosidase (KO1187), oligosaccharide‐1,6‐glucosidase (KO1182), α‐amylase (KO7405), maltose‐6′‐phosphate glucosidase (KO1232), and α‐amylase (KO1176). To investigate the metabolic response of microbes to complex fibers, we also screened the predicted abundance of KEGG entities associated with the degradation of composite fibers: β‐glucosidase (KO5349), endoglucanase (KO1179), endo‐1,4‐β‐xylanase (KO1181), β‐glucosidase (KO5350), and xylan‐1,4‐β‐xylosidase (KO1198).

We compared the relative abundance of several KEGG entities associated with chitin degradation (Borrelli et al. 2017). The following KEGG entities are related to chitin: chitinase (KO1183), chitosanases (KO1233), phospho‐chitobiase (KO1222), and chitin disaccharide deacetylase (KO3478). We also anticipated the existence of two enzymes associated with the metabolism of chitin carbon–nitrogen bonds: β‐hexosaminidase (KO1207) and α‐N‐acetylgalactosaminidase (KO1143). Kruskal‐Wallis tests and post hoc analyses (Dunn's Test) were used to compare the predicted abundance between rodent species.

## 3 Results

**FIGURE 1:**
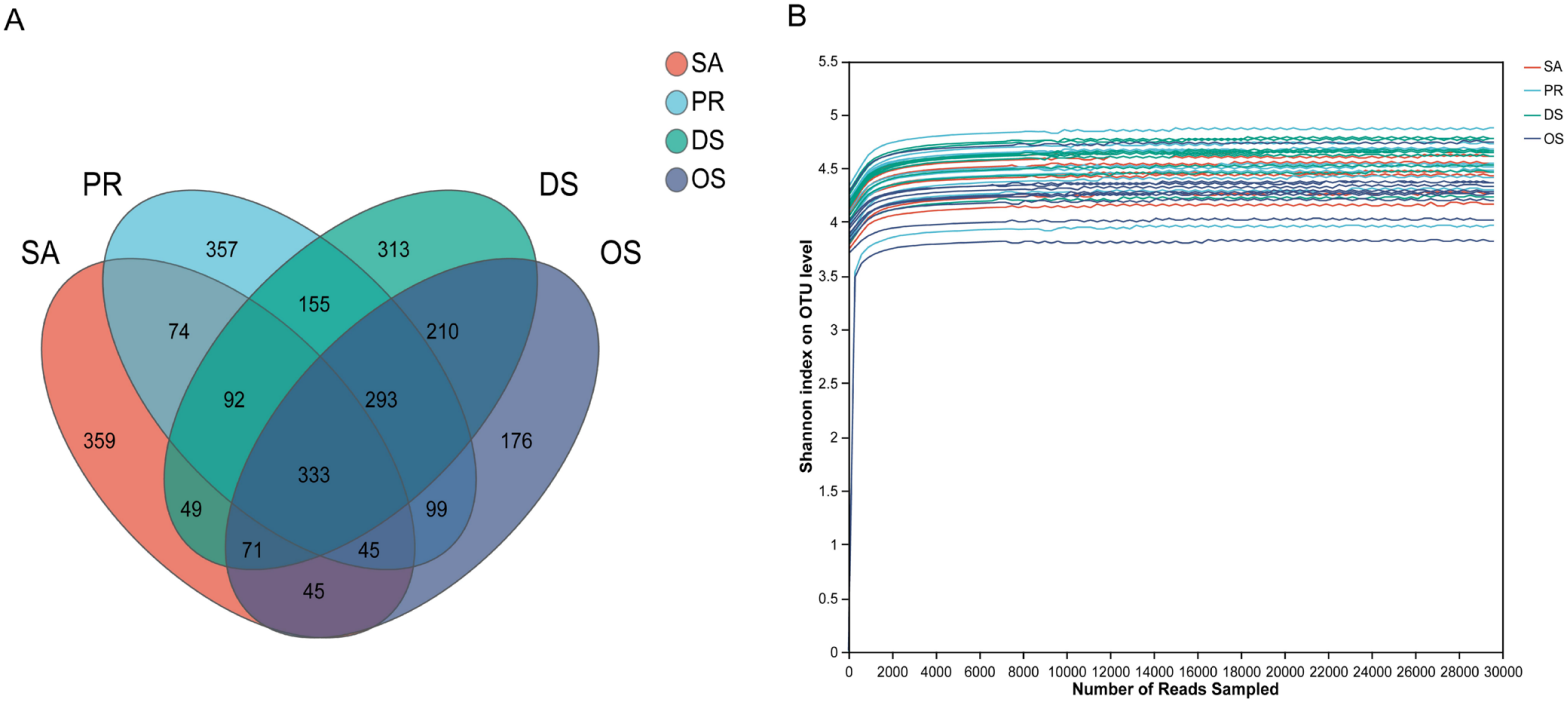
Sequencing quality and OTU analysis. (A) Venn diagram comparing the number of OTUs shared among species. The four ellipses in different colors represent four species, with numbers indicating the number of unique or shared OTUs for each species. (B) Dilution curve of gut microbiome sequences.

In this study, we analyzed the microbial composition of 7 SA fecal samples, 10 PR fecal samples, 10 DS fecal samples, and 10 OS fecal samples. The sequence range of the 37 samples was 28,759–29,013, with an average of 28,146 valid sequences per sample. A total of 1412 core OTUs were identified based on 97% sequence similarity. The DS species had an average of 483 OTUs per sample, OS had an average of 398 OTUs per sample, PR had an average of 515 OTUs per sample, and SA had an average of 450 OTUs per sample. The four species shared 333 OTUs. OS had the fewest unique OTUs, with 176, while SA had the most unique OTUs, with up to 359 (Figure 1A). This indicates that the gut microbiome has microbes that are widely distributed across these four host species as well as ones that are unique to each host's particular microbiome. Based on the rarefaction curve, the estimate of species richness is stable and unbiased, and the sequencing depth has covered almost all bacteria in the samples, making it suitable for subsequent data analysis (Figure 1B).

**FIGURE 2:**
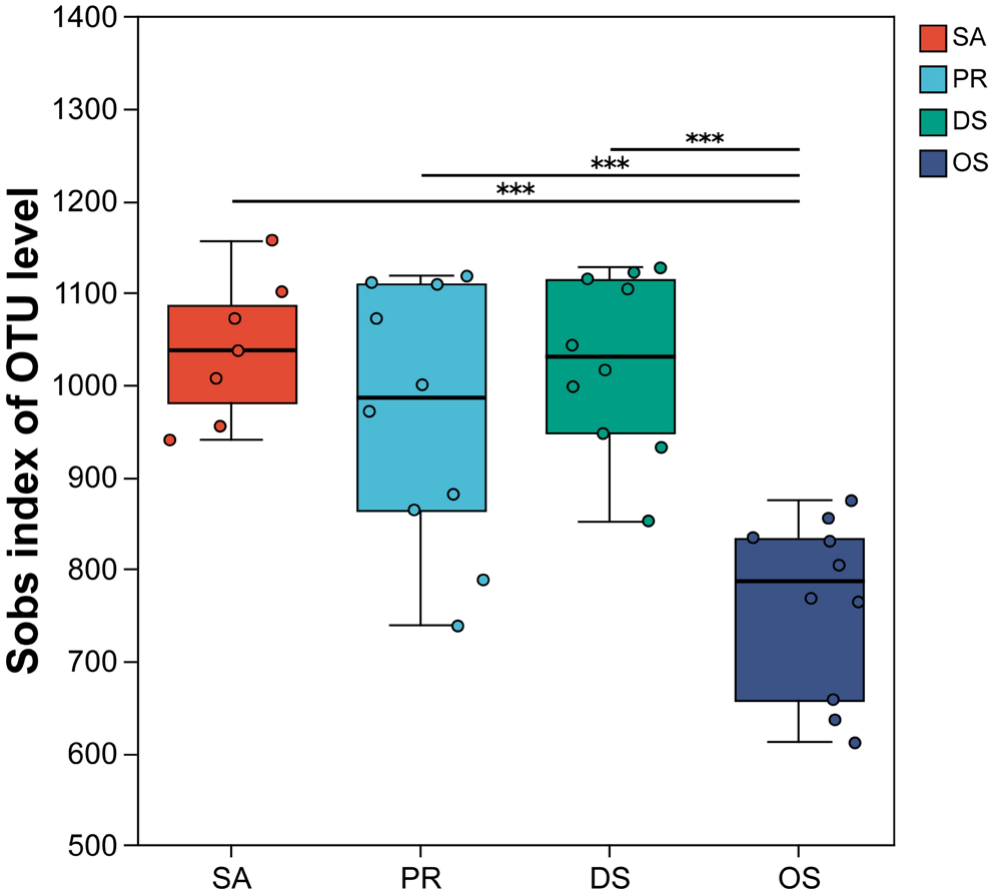
Differences in lpha diversity (Sobs diversity index) of the gut microbiome among different host species. with horizontal lines indicating the results of pairwise Kruskal–Wallis tests. Adjusted *p*‐values are represented by the following symbols: (***) for *p* < 0.001.

We calculated the Sobs index of gut microbiota for four sympatric desert rodent species to compare the alpha diversity of their gut microbial communities. Notably, the herbivorous SA exhibited higher alpha diversity in its gut microbiota, while the OS, which has a higher proportion of insectivory, had the lowest alpha diversity in its gut microbial community, showing a significant difference compared to the other three rodent species(Mean Sobs index: SA 1038, PR 965.2, DS 1026, OS 763.4) (Figure 2).

**FIGURE 3:**
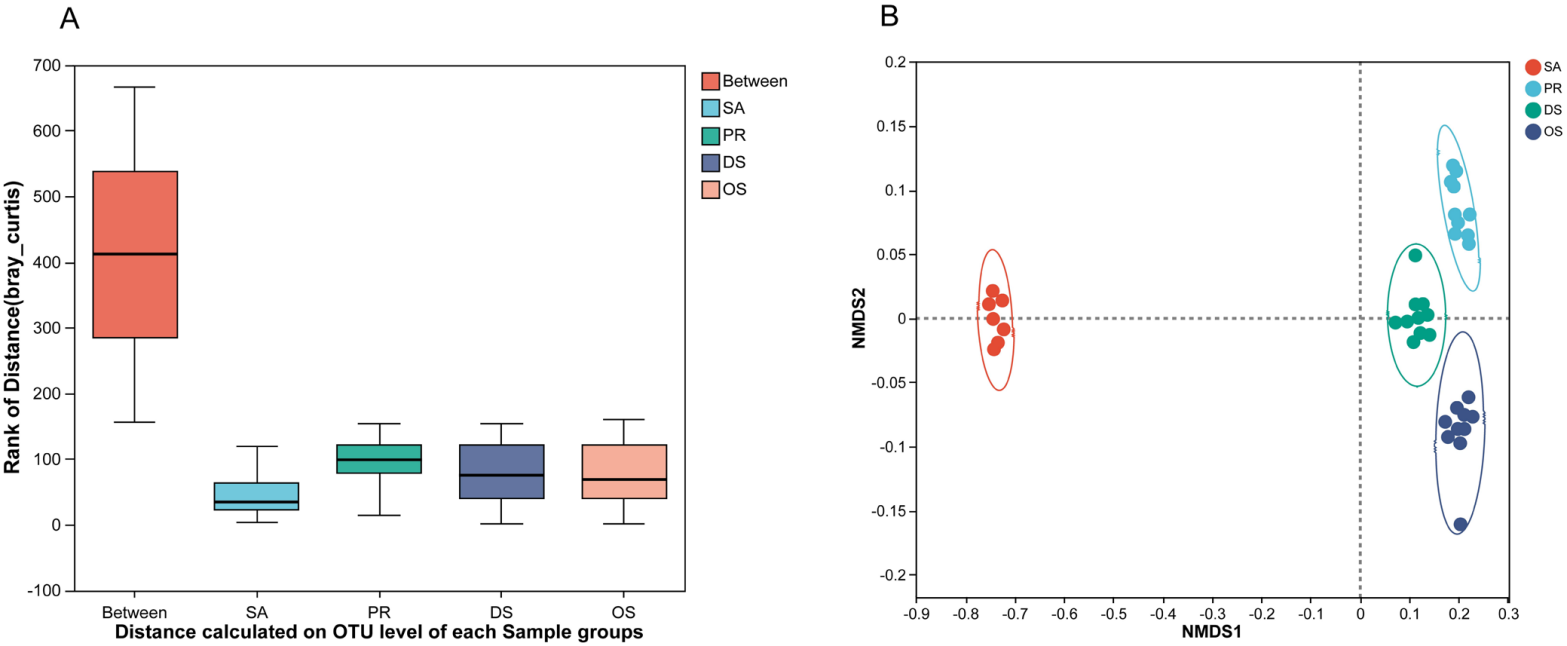
Beta diversity of the gut microbiome in rodents. (A) Boxplot of ANOSIM analysis. The x‐axis represents the values between species as well as within each species. The y‐axis represents the magnitude of the distance values. *R* values typically range from 0 to 1; higher *R* values indicate greater between‐group differences compared to within‐group differences. (B) NMDS analysis of gut microbiota in four rodent species.

Microbial communities also vary between individual hosts, so we used an ANOSIM analysis to assess how much the gut microbiomes varied within and between species. This analysis showed that the insectivore OS had greater inter‐individual variation than the other rodent species. We also found significant inter‐species differences that were much greater than intra‐species differences (Figure 3A, *r* = 0.999, *p* = 0.001, Box plot dataset range: SA Min 4, Max 119; PR Min 15, Max 153; DS Min 1, Max 154; OS Min 2, Max 160), a result that was also confirmed in the NMDS analysis, where the different species showed strong clustering. This included a clear separation of the herbivore SA microbiome from the microbiomes of the other species (Figure 3B, stress = 0.093, *p* = 0.001). These differences were statistically supported by the significant effect of host species on microbiome structure (PERMANOVA: pseudo‐F = 9.28, *p* = 0.001), indicating that these rodents possessed different microbial communities.

**FIGURE 4:**
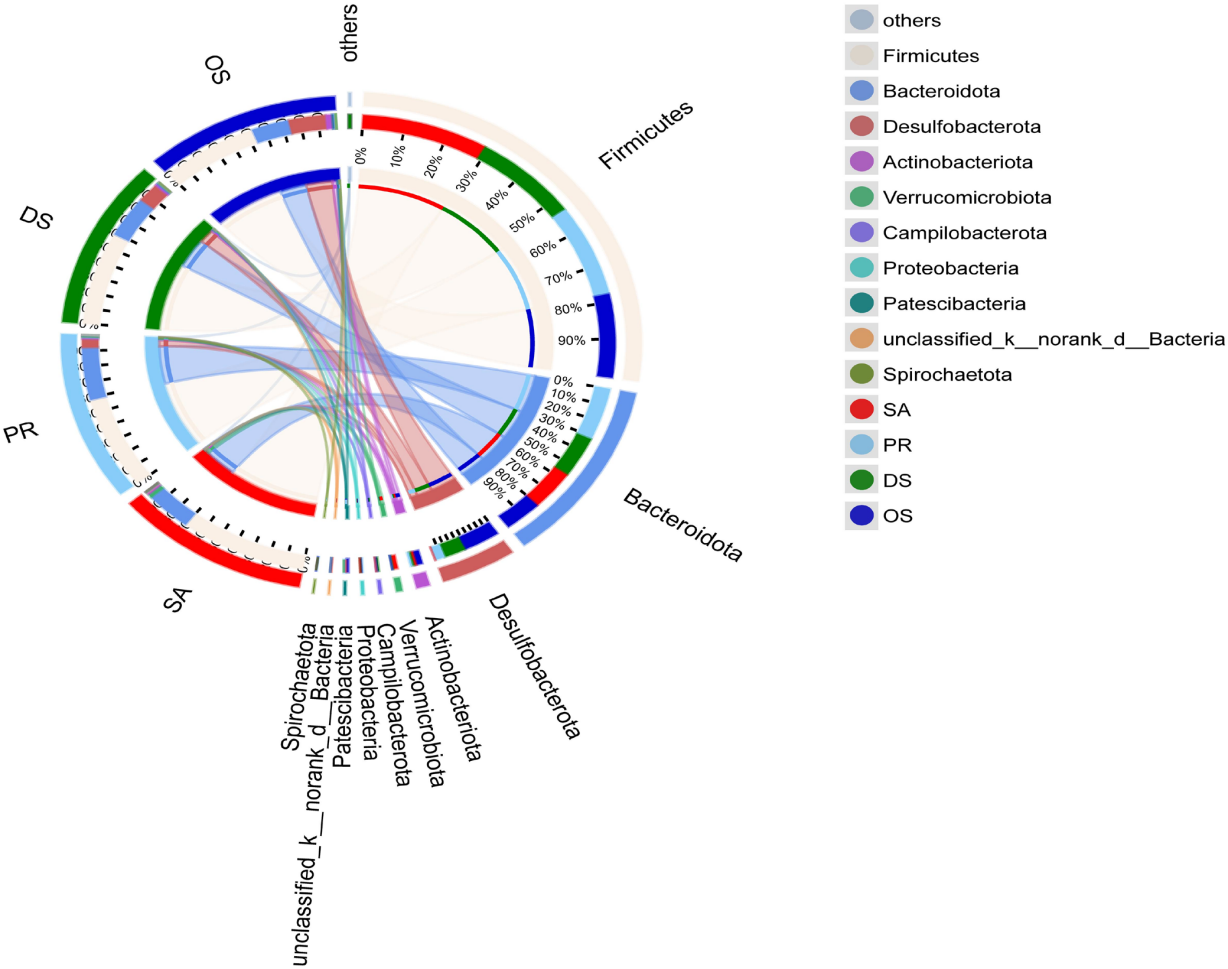
Circos plot showing the distribution of microbial taxa at the phylum level in four rodent species. The left semicircle represents the composition of species in the samples, where the outer colored bands represent the rodent host species, the inner colored bands represent the microbial taxa, and the lengths represent the relative abundance of the species in the corresponding samples. The right semicircle illustrates the distribution of different microbes across hosts at the taxonomic level, where the outer bands represent microbial taxa, the inner colored bands represent rodent host species, and the lengths represent the proportion of hosts among which the taxon is distributed.

We identified the bacterial abundance patterns at the phylum level in the gut microbiota of four rodent species. Firmicutes and Bacteroidetes were the dominant bacterial phyla across these four species; however, their abundance was lower in the gut of OS compared to SA, PR, and DS. Desulfobacterota, Patescibacteria, and Actinobacteriota were most abundant in the gut of OS; Verrucomicrobiota was most abundant in the SA gut but least abundant in the gut of granivore PR (Figure 4).

**FIGURE 5:**
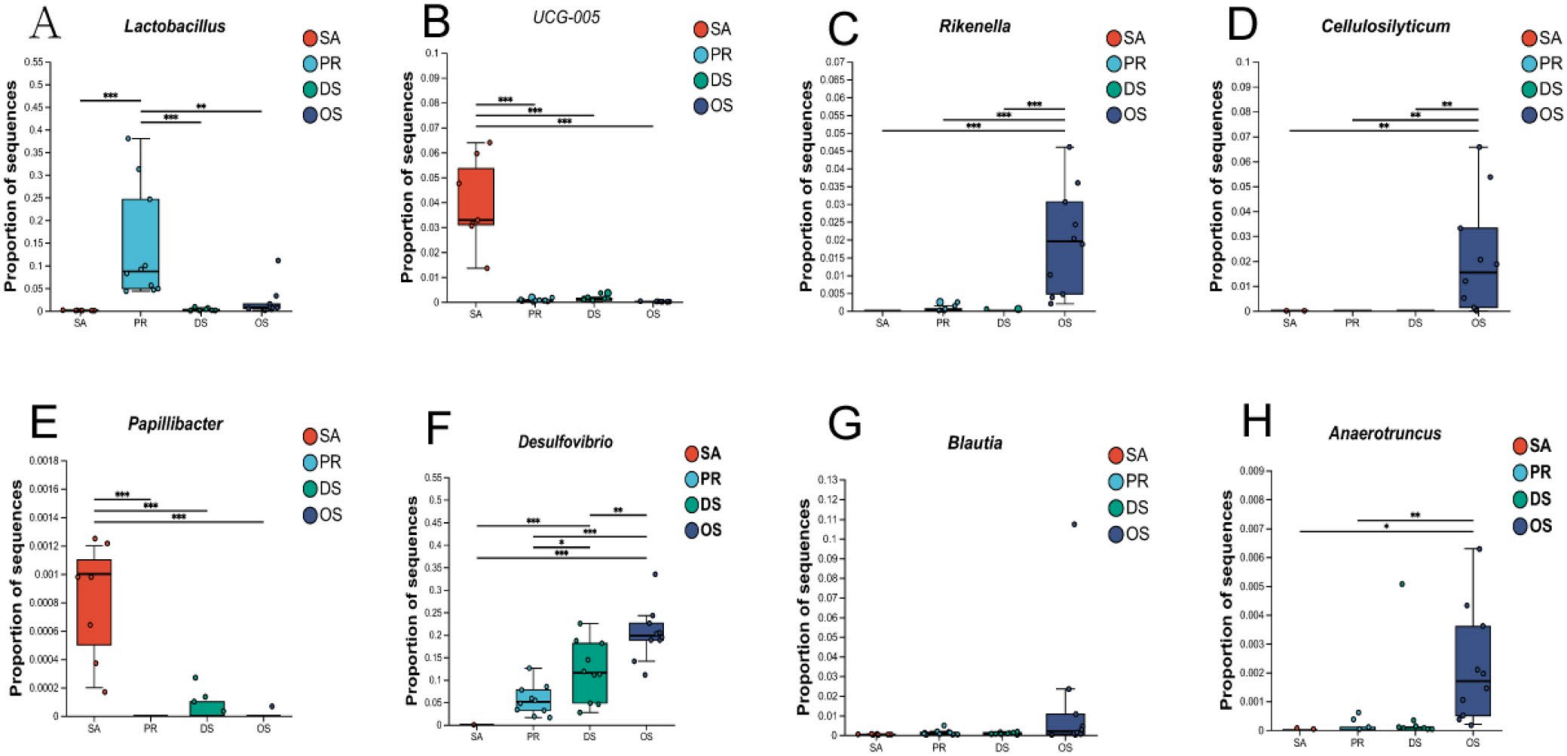
A–H represent the relative abundances of selected microbial genera: A: *Lactobacillus*, B: UCG‐005, C: *Rikenella*, D: *Cellulosilyticum*, E: *Papillibacter*, F: *Desulfovibrio*, G: *Blautia*, H: *Anaerotruncus*. Points represent data from individual rodent species, and lines represent the median. Lines with asterisks indicate the results of pairwise Kruskal–Wallis tests. Adjusted *p*‐values are denoted by the following symbols: (*) for *p* < 0.05; (**) for *p* < 0.01; (***) for *p* < 0.001.

At the genus level, we were able to identify genera that were uniquely present or absent in each rodent species studied. *Lactobacillus* (a bacterium with lactic acid fermentation capacity that converts carbohydrates into lactic acid, Figure 5A) (Hynönen and Palva 2013) was only common in the intestine of PR. *Papillibacter* (a bacterium involved in the breakdown of dietary fiber in food, Figure 5E) (Zong et al. 2023) was found only in the intestinal tracts of the herbivorous SA and the omnivorous DS. In addition, we were able to identify missing taxa in certain species. For instance, the microbial genus *UCG‐005* (Figure 5B), a bacterium associated with the degradation of fibers in herbivorous hosts, was completely absent in granivore PR and omnivorous DS and OS. *Desulfovibrio* (Figure 5F) showed a conspicuous absence in the SA gut. We also identified more bacterial genera that were significantly more abundant in the gut of the insect‐eating OS, such as *Rikenella* (Figure 5C), *Cellulosilyticum* (Figure 5D), *Blautia* (Figure 5G), and *Anaerotruncus* (Figure 5H).

In comparing the microbial OTUs detected within the dataset to those present in the PICRUSt2 database, we found that all 2901 OTUs' NSTI values (the most recent sequencing classification index) were less than 2.0, thus ensuring that no OTU was excluded from the PICRUSt2 analysis (Table S2). All OTUs' NSTI values are comparably modest (average ± standard deviation: 0.321 ± 0.194; median: 0.279). The average weighted NSTI values differed significantly by rodent species (Kruskal‐Wallis: *H* = 22.182, *p* < 0.001), with OS boasting the highest weighted NSTI value (average: 0.261) and PR exhibiting the lowest average (average: 0.194). These values are comparable to those obtained in human research; hence one should be able to predict the content of the microbiome based on the 16S rRNA gene library.

**FIGURE 6:**
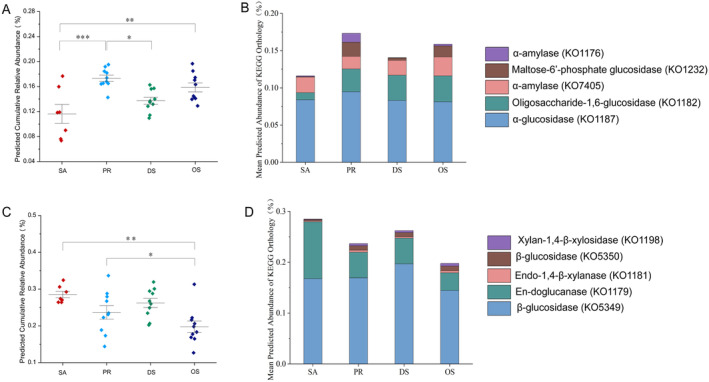
Predicted abundance of genes related to sugar metabolism. (A) Predicted abundance of genes related to monosaccharide degradation. (B) Stacked bar chart depicting the relative contributions of the top five most abundant KEGG categories related to simple sugar digestion. (C) Predicted abundance of genes related to complex fiber degradation. (D) Stacked bar chart depicting the relative contributions of the top five most abundant KEGG categories related to fiber digestion. For A and C, points represent data from individual rodent species, and lines with bars represent the mean ± standard error. Brackets with asterisks indicate the results of pairwise Kruskal‐Wallis tests. Adjusted *p*‐values are denoted by the following symbols: (*) for *p* < 0.05; (**) for *p* < 0.01; (***) for *p* < 0.001.

The microbiome of the herbivorous SA boasts the highest predicted abundance of fiber‐decomposing genes; conversely, the microbiome of the more insectivorous OS exhibits the lowest abundance of such genes. The granivore PR had the highest predicted abundance of microbial genes for metabolizing carbohydrates. When examining the genetic elements associated with the digestion of simple sugars (Figure 6A,B; Kruskal‐Wallis: *H* = 15.859; *p* < 0.001) or fibers (Figure 6C,D; Kruskal‐Wallis: *H* = 12.317; *p* < 0.01), the outcomes remain in line with our predictions. The PR has the most genes associated with the digestion of simple sugars. The herbivorous SA has the most genes related to the degradation of complex fibers, whereas the more insectivorous OS has the fewest such genes. Across rodent species, the most plentiful gene related to the digestion of simple sugars is predicted to be alpha‐glucosidase, KEGG Ontology (KO1187), and the most abundant gene associated with the complex fiber metabolism is expected to be β‐glucosidase, KEGG Ontology (KO5349) (Table S3).

**FIGURE 7:**
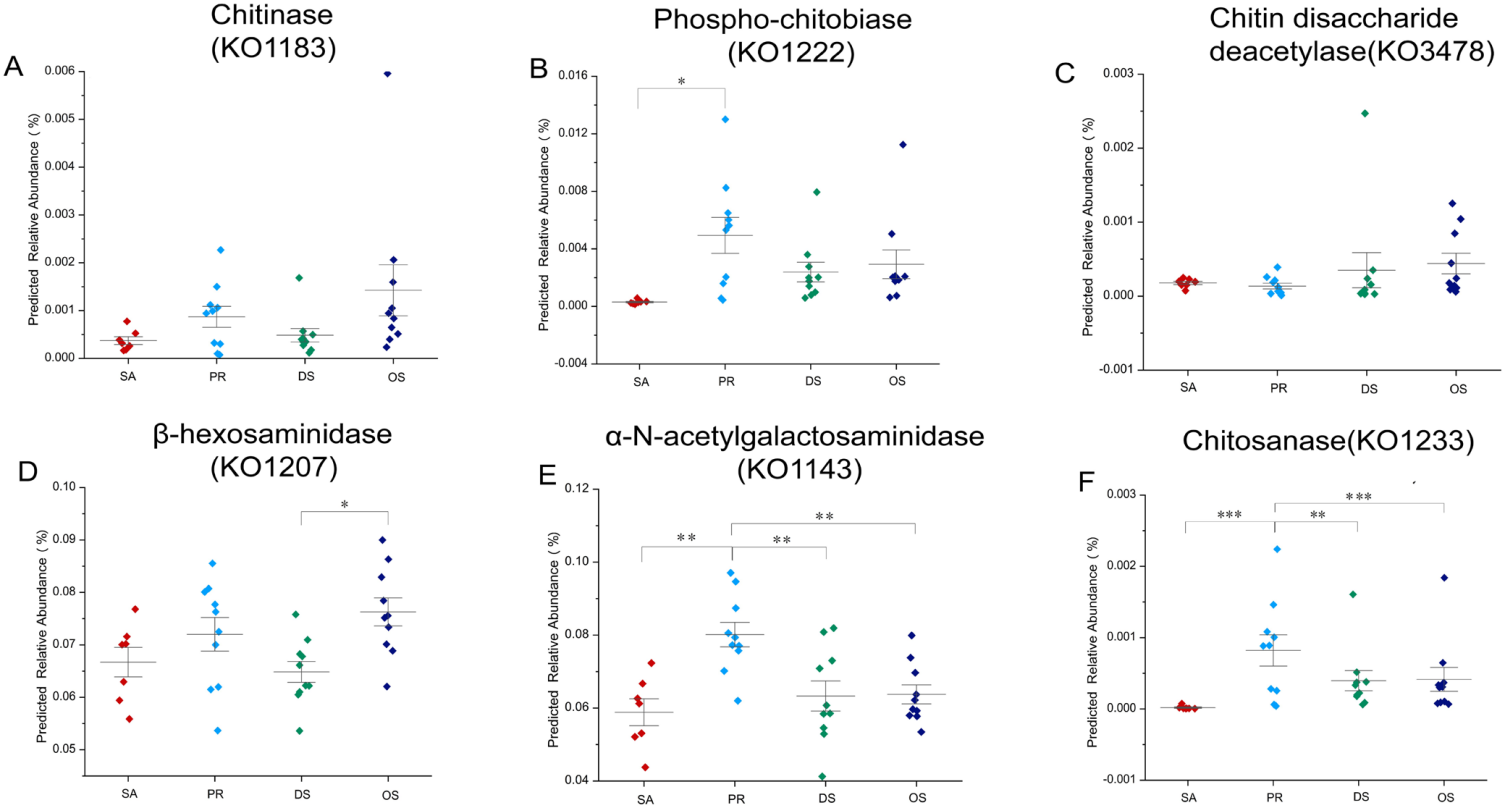
A–F represent the predicted gene abundances associated with chitin digestion: A: Chitinase (KO1183). B: Phospho‐chitobiase (KO1222). C: Chitin disaccharide deacetylase (KO3478). D: β‐hexosaminidase (KO1207). E: α‐N‐acetylgalactosaminidase (KO1143). F: Chitosanases (KO1233). Points represent data from individual rodent species, and lines with bars represent the mean ± standard error. Brackets with asterisks indicate the results of pairwise Kruskal–Wallis tests. Adjusted *p*‐values are denoted by the following symbols: (*) for *p* < 0.05; (**) for *p* < 0.01; (***) for *p* < 0.001.

Genes associated with chitin digestion are most abundant within the microbiome of the most insectivorous species, OS (Figure 7). The predicted chitinase and chitinase‐based disaccharide deacetylase abundance was higher for OS than for any of the other rodent species, at approximately two to four times higher abundance (Figure 7A,C; Kruskal‐Wallis: *H* = 17.115; all genes *p* < 0.001). It is worth noting that neither the phosphoryl chitinases nor the chitinase domain containing the acetyl group deacetylase genes are predicted to exist within the herbivorous SA, yet they are predicted to be present within the omnivorous DS (Figure 7B,C). The genes associated with the metabolic pathways of chitinase and the carbon‐nitrogen bonds within chitin varied significantly across rodent host species; however, the most insectivorous species, OS, exhibit lower predicted abundance for these categories (Figure 7E,F; α‐N‐acetyl neuraminidase KO1143, Chitinase KO1233) (Table S3).

## 4 Discussion

In this study, we employed DNA metabarcoding techniques to investigate the intestinal microbial ecology of four sympatric desert rodent species. Our focus was on the structural and functional relationships between gut microbiome composition of these species and their dietary strategies. We have demonstrated that these four desert rodent species possess distinct gut microbiome communities. This outcome mirrors the findings of other studies on wild rodents (Wang et al. 2022; Kohl et al. 2016), with the structure and composition of the gut microbiome significantly influenced by the host's evolutionary history and diet (Walter and Ley 2011; Youngblut et al. 2019). In terms of their evolutionary history, the gut microbiota of the herbivorous SA shows a clear separation from that of the other three rodent species. Among these four rodent species, the SA is the only one belonging to the suborder Scuiromorpha, while the other three species all belong to the suborder Myomorpha. The two most closely related rodent species in this study were the two jerboa species (DS and OS); these two hosts had the most similar gut microbiomes, but due to their distinct diets, their microbial communities were also markedly different. We also observed differences in the predicted abundance of genes with specific metabolic functions among these rodent species with different diets. We will now discuss two main findings regarding the gut microbiomes of these rodents: (i) the gut microbiota of herbivorous/seed‐based rodents is correlated with complex carbohydrate digestion, and (ii) the gut microbiota of predominantly insectivorous rodents is associated with chitinase. Finally, we will delve into the merits and limitations of the DNA metabarcoding techniques in the study of microbiota and the pursuit of scientific advances.

### 4.1 Feeding Habits of Herbivorous/Granivore Rodents and the Degradation of Carbohydrates

We found that SA, the herbivore, has the most diverse gut microbiome out of the rodents studied. Prior research has found that, in mammals, the gut microbiomes of herbivores are far more diverse than those of mammals with other diets (Fu et al. 2021; Kobayashi et al. 2020). It is believed that this higher diversity is the result of the presence of fibers in plant tissue. These fibers are difficult for animals to directly digest and absorb (Wilson and Kennedy 1996; Ramamoorthy et al. 2015), so the diversity of gut microbiota among herbivores like SA may aid in their food digestion. Gut microbial diversity is also correlated with the host's body size, with larger animals typically containing greater gut microbial diversity (Ley et al. 2008; Rojas et al. 2021). Interestingly, SA is also the largest of our study species (Table 1), which could be an additional contributing factor for the higher microbial diversity in this species.

We also discovered that, in line with our predictions, the herbivore SA has the highest abundance of cellulose‐decomposing genera—*UCG‐005*—and the highest predicted abundance of genes associated with complex fiber digestion. However, the predicted abundance of genes associated with monosaccharide digestion is highest within the intestinal tract of PR. The predicted abundance of genes related to complex fiber digestion was elevated in PR, defying our anticipated pattern. We expect that genes associated with monosaccharides are also most plentiful within the herbivore SA, due to cellulose needing to be fermented into volatile fatty acids to be absorbed and utilized, and monosaccharides being important intermediates in cellulose fermentation (Lynd et al. 2002). However, the predicted abundance of genes related to monosaccharide digestion in the gut of the SA is not high. This may be because monosaccharides, as intermediates in cellulose fermentation, do not remain in the gut in large quantities or for extended periods (Hungate 1944). The higher abundance of genes related to monosaccharide and fiber degradation in the gut of the PR is due to the large amount of starch present in plant seeds, as well as the fibrous, indigestible seed coats (Sfiligoj Smole et al. 2013; Mudgil and Barak 2013). In our analysis of the stomach contents of the PR, we observed a large amount of plant seed coats. Therefore, the PR relies on its gut microbiota to digest these starches and celluloses, thus maintaining its digestive function. In addition, research on mammal digestive processes has contrasted the strategies of maximum yield and maximum velocity. Animals that exhibit maximum yield generally show longer intestinal passage times, whereas those that maximize velocity are characterized by rapid digestive tract movement (Clements and Rees 1998; Choat et al. 2004). Maximum yield is typically associated with a larger body size, which increases the amount of time that food is located within the intestine (Cummings and Overduin 2007). The small body size of PR implies that it is expected to maximize velocity rather than yield, which would require its microbiome to be capable of utilizing simple sugars. Of course, this hypothesis requires additional investigation and validation.

### 4.2 Feeding Habits of Insectivorous Rodents and the Decomposition of Chitin

*O. sibirica* , the most insectivorous species studied, possesses a unique gut microbiome distinct from that of the other rodents studied, with the lowest species diversity in this study. This is more common among mammals that consume higher nutrient levels, so perhaps this is due to its relatively narrow diet with a high proportion of insects (Bolnick et al. 2014; Huang, Sham, et al. 2020). The diets of insectivorous animals are generally high in protein and chitin (Shah et al. 2022; Gorbunova and Zakharov 2021), and the relative scarcity of carbohydrates might restrict the survival and reproduction of certain microorganisms. The stomachs of insectivores are typically more acidic (Acosta‐Estrada et al. 2021; Dossey et al. 2016), which could also be detrimental to microbes, thus possibly contributing to why OS intestinal microbiome has relatively low diversity.

Upon further examination of the microbial community of OS, several distinctive patterns emerge. For instance, the genus *Anaerotruncus* was detected exclusively within the guts of OS. Members of this genus may possess anti‐inflammatory properties, which promote the health of intestinal mucosa and mitigate chronic inflammation (Huang, Li, et al. 2020; Mu et al. 2021). Some studies have indicated that insectivorous mammals are prone to intestinal inflammation (Jiminez et al. 2015). Therefore, the presence of Anaerotruncus may help the host resist inflammation and thus maintain its physiological health. Furthermore, the genera *Rikenella* and *Blautia* are also abundant within the intestinal microbiota of insectivores. These microbial communities are ubiquitously present within the intestinal tracts of animals, yet their specific role within the host's intestinal ecosystem remains unclear. They have been found to exert a profound influence on the host's physiological processes, including energy metabolism, communication of signals, and immune responses (Benítez‐Páez et al. 2020; Liu et al. 2021).

Interestingly, we discovered that the genes associated with chitin digestion were predicted in the intestinal tracts of all four rodent species, including the herbivorous SA, which may be due to the propensity of rodents to opportunistically feed on insects (Vaughan 1974). However, the species that eats the most insects, OS, possesses three genes with higher predicted abundance related to chitin degradation. Chitinase (a hydrolyzing enzyme, utilized for the degradation of long‐chain chitin), chitin disaccharide deacetylase (an enzyme catalyzing the removal of acetyl groups in chitin degradation), and β‐hexosaminidase (a gene category related to the metabolism of carbon–nitrogen bonds in chitin) are intimately tied to the degradation of chitin (Schrempf 2001). Surprisingly, we found that the predicted abundance of genes related to chitin degradation in PR's gut was also high., which may be associated with PR's food sources. Our sampling was conducted in August, a period when plant seeds in desert regions mature. However, before the seeds mature, PR may consume a certain proportion of insects, as reported in other studies (Wang et al. 2001), which could lead to the higher abundance of genes related to chitin degradation in its gut. Furthermore, we propose that the higher abundance of chitin degradation‐related genes in PR's gut is closely related to its primary food source, seeds. Plant seeds often contain large amounts of endophytic fungi (Guan et al. 2005), the cell walls of which are primarily composed of chitin (Feofilova 2010). When PR consumes seeds, it not only ingests the seeds themselves but also the microorganisms associated with them. To efficiently digest these microorganisms, PR's gut microbiota may need to produce more chitin‐degrading enzymes. Additionally, PR's small body size suggests that it may be a rate‐maximizing strategist according to digestive theory. Such animals prioritize maximizing nutrient absorption rate over yield, enabling efficient energy acquisition during rapid food passage (Choat et al. 2004). Therefore, PR's gut microbiota likely needs to rapidly degrade chitin and convert it into nutrients that can be utilized by the host. However, the abundance of microbial functions does not necessarily result in increased functional capacity. Microbes may be digesting chitin for their own benefit without providing any benefits for the host; this hypothesis requires further investigation and validation. In addition, we have observed that the predicted abundances of chitosanases and another gene related to the metabolism of carbon–nitrogen bonds in chitin were not enriched in the gut of OS. Therefore, chitin metabolism within animal hosts requires increased attention.

### 4.3 Constraints, Limitations, and Scientific Validity

Our study on the gut microbiomes of four rodent species is primarily descriptive due to the constraints of our sample size—the limitations of our samples are determined by species, population, and individual differences. Rodents' diets may vary over time and space (Davis and Pineda‐Munoz 2016; Smith and Lyons 2011), yet these fluctuations are not measured in our sample. Moreover, as the dietary diversity within our sample varying by species only rather than within species, this limits our ability to discern the difference between the effects of the host's diet and other effects that vary by species. However, our research was a naturalistic study and also employed advanced sequencing technologies, thus ensuring the natural validity of our measures.

DNA metabarcoding techniques are commonly employed molecular biology methods for investigating microbial community structure. Despite their significant advantages in characterizing microbial diversity, they do possess certain limitations. Firstly, the sequence of DNA metabarcoding provides only relatively low‐resolution data, so in some cases the precise microbial strains cannot be distinguished. Secondly, due to the varying copy numbers of DNA metabarcoding genes, there exists a bias towards taxa with a higher copy number, which may lead to an underestimation or overestimation of microbial abundance (de la Fuente et al. 2014; Regueira‐Iglesias et al. 2023). We used the PICRUSt2 method to infer the functional characteristics of microbial communities through the anticipation of functional genomes. Although PICRUSt2 offers valuable insights into function prediction, its predictions are still subject to several possible biases. Firstly, this method relies on the accuracy of the DNA metabarcoding and the integrity of the database. Subsequently, because of any possible inaccuracies in microbial genomes, the predicted functions of PICRUSt may not thoroughly reflect reality (Li, Li, et al. 2022; Gao et al. 2021). Despite their inherent constraints, these methods continue to be essential tools in the field of microbial community structure and function. These limitations must be kept in mind, but we believe that despite them, these methods allow for the discovery of reasonably accurate information. Advanced sequencing techniques and methodologies do indeed furnish a more plentiful and precise dataset, yet they can never completely escape such limitations.

The benefits of DNA metabarcoding techniques lie in their ability to resolve a multitude of issues by leveraging a single dataset. Despite the constraints imposed on the depth of our sequencing, this paper's data contributes to our understanding of how the gut microbiome and its potential functions vary across hosts. Therefore, the sequencing technology of DNA metabarcoding is a useful tool that can lead to new discoveries in various topics. For instance, we have discovered the potential adaptations of the intestinal microbiota of herbivorous rodents and rodents with higher proportions of insects in their diet. Furthermore, we have investigated individual variation, enhancing our understanding of the gut microecology of rodents with different dietary strategies, thereby opening up new avenues for research into the microbial ecology of these wild rodents.

## 5 Conclusion

We observed significant differences in the gut microbial composition of four species of rodents living in the same habitat but each consuming different diets. These differences in gut microbiota may arise as adaptations to their distinct diets and digestive needs, thereby enhancing their ability to live within the same ecosystem.

## Author Contributions

**Dongyang Chu:** data curation (lead), formal analysis (equal), investigation (equal), methodology (lead), visualization (lead), writing – original draft (lead), writing – review and editing (lead). **Haoting Zhang:** conceptualization (supporting), software (supporting). **Zhenghaoni Shang:** conceptualization (equal), methodology (supporting), software (supporting), visualization (equal). **Nan Liu:** conceptualization (equal), investigation (equal), methodology (equal), supervision (equal), writing – original draft (equal). **Heping Fu:** funding acquisition (supporting), project administration (equal), supervision (lead), writing – review and editing (supporting). **Shuai Yuan:** conceptualization (equal), data curation (supporting), investigation (lead), methodology (equal), project administration (lead), resources (equal), supervision (lead), writing – review and editing (equal).

## Conflicts of Interest

The authors declare no conflicts of interest.

## Supporting information


Data S1.

**Table S1.** Proportions of food types for four rodent species.

Table S2.

Table S3.

## Data Availability

The original data are available in the NCBI data repository: https://www.ncbi.nlm.nih.gov/biosample?LinkName=bioproject_biosample&from_uid=1083208. The original data are stored in the NCBI data repository for private peer review: https://www.ncbi.nlm.nih.gov/biosample?LinkName=bioproject_biosample&from_uid=1083208.
