# Supplementary material for: Gut Microecology of Four Sympatric Desert Rodents Varies by Diet
Source: Ecol Evol. 2025 Feb 27;15(3):e70992. doi: 10.1002/ece3.70992 (PMC11868701; doi:10.1002/ece3.70992)
Supplement: Supplementary file 1 — Data S1. [file ECE3-15-e70992-s002.docx]

**PCR Amplification**

Primer Target Region: 16S V3-V4 Region

Forward Primer 338F: ACTCCTACGGGAGGCAGCAG

Reverse Primer 806R: GGACTACHVGGGTWTCTAAT

**PCR Amplification**

Primer Target Region: 16S V3-V4 Region

Forward Primer 338F: ACTCCTACGGGAGGCAGCAG

Reverse Primer 806R: GGACTACHVGGGTWTCTAAT

(1) PCR Reaction System:

5×FastPfu Buffer: 4 µL

2.5 mM dNTPs: 2 µL

Forward Primer (5 µM): 0.8 µL

Reverse Primer (5 µM): 0.8 µL

FastPfu Polymerase: 0.4 µL

BSA: 0.2 µL

Template DNA: 10 ng

Add ddH2O to a total volume of 20 µL

(2) PCR Reaction Parameters:

a. 1× (3 minutes at 95℃)

b. [Number of cycles] × (30 sec at 95℃; 30 sec at [Annealing Temperature]℃; 45 sec at 72℃)

c. 10 minutes at 72℃, 10℃ until halted by user

PCR Product Identification, Purification, and Quantification

PCR Product Identification:

Perform three PCR replicates for each sample and mix the three replicates.

Use 2% agarose gel electrophoresis to detect the products.

**PCR Product Purification**

**Purify the PCR product using the AxyPrep DNA Gel Extraction Kit. The specific steps are as follows:**

1.Under UV light, cut the agarose gel containing the target DNA. Use a paper towel to absorb any liquid on the gel surface and cut the gel into small pieces. Weigh the gel, with the weight considered as the gel volume (100 mg = 100 µL volume).

2.Add 3 volumes of Buffer DE-A to the gel. Mix and heat at 75℃, intermittently mixing until the gel is completely melted.

3.Add 0.5 volumes of Buffer DE-B and mix thoroughly.

4.Transfer the mixture from step 3 to a DNA preparation column and centrifuge at 10,000 rpm for 1 minute. Discard the filtrate.

5.Place the preparation column back into a 2 mL collection tube. Add 500 µL of Buffer W1 and centrifuge at 12,000 rpm for 30 seconds. Discard the filtrate.

6.Place the preparation column back into the 2 mL collection tube. Add 700 µL of Buffer W2 and centrifuge at 12,000 rpm for 30 seconds. Discard the filtrate. Repeat the wash with another 700 µL of Buffer W2 and centrifuge at 12,000 rpm for 1 minute.

7.Place the preparation column back into the 2 mL collection tube and centrifuge at 12,000 rpm for 1 minute.

8.Place the preparation column into a clean 1.5 mL microcentrifuge tube. Add 25-30 µL of deionized water to the center of the preparation membrane. Let it sit at room temperature for 1 minute, then centrifuge at 12,000 rpm for 1 minute to elute the DNA.

**PCR Product Quantification and Normalization**

Quantify the PCR products using the Quantus™ Fluorometer. Mix the samples in appropriate proportions according to the sequencing requirements for each sample.

**Construction of PE Library and Illumina Sequencing**

**Miseq Library Construction**

**Use the NEXTFLEX Rapid DNA-Seq Kit for library construction with the following steps:**

1.Adapter ligation;

2.Use magnetic beads to remove self-ligated adapter fragments;

3.Enrich the library template using PCR amplification;

4.Recover the PCR products with magnetic beads to obtain the final library.

**Illumina Sequencing**

**Perform sequencing using Illumina's Miseq PE300 (Shanghai Majorbio Bio-Pharm Technology Co., Ltd.) with the following steps:**

1.One end of the DNA fragment is complementary to the primer bases and is fixed on the chip;

2.The other end randomly complements with another nearby primer and is also fixed, forming a "bridge";

3.PCR amplification produces DNA clusters;

4.Linearize the DNA amplicons to single strands;

5.Add modified DNA polymerase and four types of fluorescently labeled dNTPs, synthesizing one base per cycle;

6.Scan the surface of the reaction plate with a laser to read the nucleotide incorporated in the first cycle for each template sequence;

7.Chemically cleave the "fluorescent group" and "terminator group," restoring the sticky 3' end to continue synthesizing the next nucleotide;

8.Collect the fluorescent signal results from each cycle to determine the sequence of the template DNA fragments.
